# Aerobic Exercise Prevents Chronic Inflammation and Insulin Resistance in Skeletal Muscle of High-Fat Diet Mice

**DOI:** 10.3390/nu14183730

**Published:** 2022-09-10

**Authors:** Nan Li, Haiyan Shi, Qiaofeng Guo, Yanming Gan, Yuhang Zhang, Jiajie Jia, Liang Zhang, Yue Zhou

**Affiliations:** 1 Department of Exercise Physiology, Beijing Sport University, Beijing 100084, China; 2 School of Strength and Conditioning Training, Beijing Sport University, Beijing 100084, China; 3 Key Laboratory of Physical Fitness and Exercise, Ministry of Education, Beijing Sport University, Beijing 100084, China

**Keywords:** insulin resistance, inflammation, aerobic exercise, skeletal muscle, NF-κB pathway

## Abstract

Obesity is commonly accompanied by chronic tissue inflammation and leads to insulin resistance. Aerobic exercise is an essential treatment for insulin resistance and has anti-inflammatory effects. However, the molecular mechanisms of exercise on obesity-associated inflammation and insulin resistance remain largely unknown. Here, we evaluated the effects of aerobic exercise on inflammation and insulin resistance in skeletal muscles of high-fat diet (HFD) mice. Male C57BL/6J mice were fed a high-fat diet or a normal diet for 12 weeks, and then aerobic training was performed on a treadmill for 8 weeks. Body weight, fasting blood glucose, food intake levels, and glucose and insulin tolerance were evaluated. The levels of cytokines, skeletal muscle insulin resistance, and inflammation were also analyzed. Eight weeks of aerobic exercise attenuated HFD-induced weight gain and glucose intolerance, and improved insulin sensitivity. This was accompanied by enhanced insulin signaling. Exercise directly resulted in a significant reduction of lipid content, inflammation, and macrophage infiltration in skeletal muscles. Moreover, exercise alleviated HFD-mediated inflammation by suppressing the activation of the NF-κB pathway within skeletal muscles. These results revealed that aerobic exercise could lead to an anti-inflammatory phenotype with protection from skeletal muscle insulin resistance in HFD-induced mice.

## 1. Introduction

Obesity is becoming a global epidemic considering that its incidence increases yearly and that it increases the health burden of associated complications of insulin resistance (IR) and diseases such as cardiovascular disease [1]. Importantly, IR is a key feature of obesity and type 2 diabetes mellitus (T2DM). Chronic inflammation plays a causal role in the development of IR [2,3], which is associated with a variety of tissues, including adipose tissue, skeletal muscle, liver, pancreas islet, and even the brain [4,5]. However, the mechanisms underlying IR-linked inflammation remain poorly understood.

As the largest metabolic organ and the most prominent site of insulin-mediated glucose uptake in humans, the skeletal muscle plays an important role in glucose and lipid homeostasis [6]. It is well-established that skeletal muscle myocytes express and secrete numerous cytokines, such as interleukin-6 (IL-6) and tumor necrosis factor-alpha (TNF-α). Most myokines are regulated mainly by exercise and muscle function and are involved in glucose and lipid metabolism and inflammation. Surprisingly, immune cells, especially macrophages, may induce myocyte inflammation, adversely regulate myocyte metabolism, and contribute to insulin resistance via paracrine effects [7]. TNF-α plays a critical role in inflammation and the development of IR [8,9]. TNF-α exerts proinflammatory effects mainly by activating the nuclear factor kappa-B (NF-κB) and c-Jun N-terminal kinase (JNK) pathways [10,11]. The activation of NF-κB in obesity can increase inflammation in macrophages, adipocytes, and muscle [12,13]. Moreover, extensive research has shown that the activation of inflammatory pathways gives rise to the development of IR [14]. Thus, the suppression of chronic inflammation in skeletal muscle may be one of the protective strategies against IR.

As a crucial part of the prevention and treatment of diabetes and metabolic diseases, physical exercise can reduce inflammation and improve IR. Regular exercise can improve insulin signaling in skeletal muscle and promote insulin sensitivity [15,16,17,18]. Moreover, exercise has the potential to modulate inflammatory processes by affecting specific inflammatory signaling pathways that can interfere with the signaling pathways of glucose uptake [19]. Previous studies have confirmed that exercise modulates the NF-κB pathways and improves insulin sensitivity in skeletal muscle [20,21]. These taken together, it is clear that chronic inflammation plays a fundamental role in the development of IR, and exercise may improve IR and glucose metabolism by reducing inflammation in skeletal muscle.

Here, we hypothesized that exercise-induced improvement in insulin sensitivity of skeletal muscle may be related to decreasing immune cell infiltration and attenuating NF-κB-related inflammation in high-fat diet (HFD) mice. We examined the effects of aerobic exercise on IR and chronic inflammation in HFD-induced obese mice, as well as the related role of the NF-κB pathway and insulin signaling pathway.

## 2. Materials and Methods

### 2.1. Animals and Diets

Five-week-old male C57BL/6J mice were obtained from Beijing Huafukang Laboratory Animal Technology Co., Ltd. (Beijing, China) and were housed in a controlled room at 22–25 °C on a 12 h day/night cycle with free access to food and water. After 5 days of acclimation, the mice were randomly divided into two groups. The normal control group (NC, *n* = 20) continued eating regular food (3.87 kcal/g, 2.79% energy as fat, 1025, Huafukang), while the high-fat diet (HFD, *n* = 30) group was fed with a high-fat diet (5.24 kcal/g, 60% energy as fat, H10060, Huafukang). IR was diagnosed if the AUC during GTT of the HFD group was 1.2 times higher than that of the NC group. After 12 weeks of diet, the mice were randomly allocated into the following four groups: normal sedentary (NS, *n* = 8), normal exercise (NE, *n* = 8), HFD sedentary (HS, *n* = 8), and HFD exercise (HE, *n* = 8). Body weight and fasting blood glucose concentration were measured once a week. Blood was collected from the tail vein.

### 2.2. Exercise Protocol

All mice in the exercise groups were tested for exercise capacity before beginning the exercise training sessions. In the exercise capacity test, the treadmill speed was initially set at 10 m/min. Exercise capacity was determined by the graded increase in treadmill speed (3 m/min every 3 min) with an incline of 0% until exhaustion. The animals were adapted to the procedure by increasing the duration and speed of running for 3 days consecutively before beginning the exercise training sessions. In accordance with the classical treadmill exercise model [22], aerobic exercise training was performed on a treadmill with an incline of 0°, 15–20 m/min (55–65% VO_2_max), 60 min/day, 5 days/week, for 8 weeks. Mice in the sedentary groups were placed on the treadmill for the same duration as the exercise-trained mice.

### 2.3. Glucose Tolerance Test and Insulin Tolerance Test

**Figure 1:**
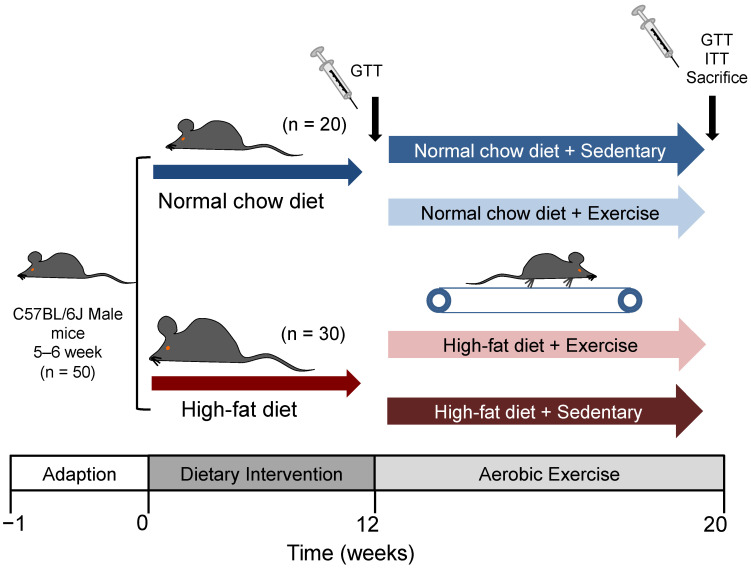
Experimental design of the study. GTT, glucose tolerance test; ITT, insulin tolerance test.

After an overnight fast, the mice received an intraperitoneal injection of glucose (2 g/kg). Blood samples were obtained from the tail vein at 0, 30, 60, 90 and 120 min after the glucose challenge. The mice were fasted for 4 h and injected with insulin (0.5 U/kg) for the insulin tolerance test (ITT) (Figure 1). Blood glucose concentrations were measured by a glucometer (Roche Ltd., Basel, Switzerland).

### 2.4. Sample Collection

After 20 weeks of intervention, the mice were euthanized using isoflurane inhalation after fasting for 12 h and 48 h at the end of the last exercise session. Blood samples were collected after removal of the eyeball, and then the mice were killed by cervical dislocation. After that, the whole blood samples were allowed to clot by leaving them undisturbed at room temperature for 30 min. Then, the clot was removed to obtain the serum by centrifugation at 3000 rpm for 15 min at 4 °C, and the supernatant was taken as experimental serum. The skeletal muscles, adipose tissue, and liver were dissected and weighed quickly. Serum and tissues were stored at −80 °C for subsequent use.

### 2.5. Serum Analysis

The serum levels of triglyceride (TG), total cholesterol (TC), high-density lipoprotein (HDL), and low–-density lipoprotein (LDL) were measured with appropriate kits (Njjcbio, Nanjing, China) using a clinical chemistry analyzer (AU5800, Beckman, Sacramento, CA, USA). The fasting insulin (FINS) (EZRMI-13K, Millipore, Billerica, MA, USA), tumor necrosis factor-α (TNF-α), interleukin-1β (IL-1β), and interleukin-10 (IL-10) (AD3051Mo, AD3364Mo, and AD2837Mo, Andygene, Beijing) levels were measured using enzyme-linked immunosorbent assay (ELISA) kits. Homeostasis model assessment of insulin resistance (HOMA–-IR) was determined using the following equation: HOMA–-IR = [FINS (mU/L) × FPG (mmol/L)]/22.5.

### 2.6. Morphometric Analysis

The left gastrocnemius muscles were dissected rapidly, and the muscle tissues were weighed and fixed with 4% polyaldehyde for 24 h. Serial 8–10 μm thick transverse sections were stained with hematoxylin and eosin. The right gastrocnemius muscles were frozen in optimal cutting temperature compound, sectioned with a cryostat into 10 μm thick sections, and stained with Oil Red O. The average areas of the myofibers and lipid deposits in each of the images were measured using the image analysis software Image-Pro Plus 6.0.

### 2.7. Western Blot Analysis

The protein concentrations in the quadriceps muscles were detected using the BCA protein assay kit. The total protein (40 μg or 80 μg) was separated on 10% SDS-polyacrylamide gels and transferred to polyvinylidene fluoride membranes. The immunoblots were incubated at 4 °C overnight with primary antibodies, including inhibitor of kappa B (IκB-α) (1:10,000, 9242), NF-κB (1:2000, 8242), insulin receptor substrate 1 (IRS-1) (1:500, 2382), phosphoinositide 3-kinase (PI3K p85, 1:5000, 4292), protein kinase B (AKT) (1:10,000, 9272), phosphorylated Akt (p-Akt, Ser 473, 1:1000, 4060), and glucose transporter type 4 (GLUT4) (1:1000, 2213), all of which were obtained from Cell Signaling Technology, Inc., Beverly, MA, USA. Glyceraldehyde-3-phosphate dehydrogenase (GAPDH, YM3029, Immunoway, Plano, TX, USA) was used as a loading control (1:20,000). The process was followed by incubation with the secondary antibodies at room temperature for 2 h. The anti-rabbit antibody-HRP conjugate was used at 1:10,000 as the secondary antibody. After incubation, the blots were detected with enhanced chemiluminescence. Protein bands were captured using an Azure Biosystems C300 imaging system (Azure Biosystems, Dublin, CA, USA), and optical densities were quantified using ImageJ (RRID: SCR_003070) software.

### 2.8. Quantitative Real-Time Polymerase Chain Reaction Analyses

Total RNAs were isolated from the quadriceps muscles using TRIzol RNA isolation reagent (Invitrogen, Carlsbad, CA, USA) and subjected to conventional cDNA synthesis and real-time polymerase chain reaction (PCR). The levels of gene expression were calculated based on the 2^ΔΔ^CT method. The primer sequences and expected product size for the genes amplified are listed in Table 1 (all primers were synthesized by Sangon Biotech Co., Ltd., Shanghai, China).

### 2.9. Statistical Analysis

All the results are presented as the mean ± SD. Statistical analyses were performed using SPSS statistical software (v.26, SPSS Institute, Chicago, IL, USA) and GraphPad Prism software (v.7, GraphPad software Inc., San Diego, CA, USA). The repeated-measures analysis of variance (ANOVA) was used to analyze the differences in body weight, fasting blood glucose, and food intake. Grouped data were examined by two-way ANOVA with Bonferroni’s multiple-comparison test. A *p* value < 0.05 was considered statistically significant.

## 3. Results

### 3.1. Aerobic Exercise Attenuated HFD-Induced Adiposity

**Figure 2:**
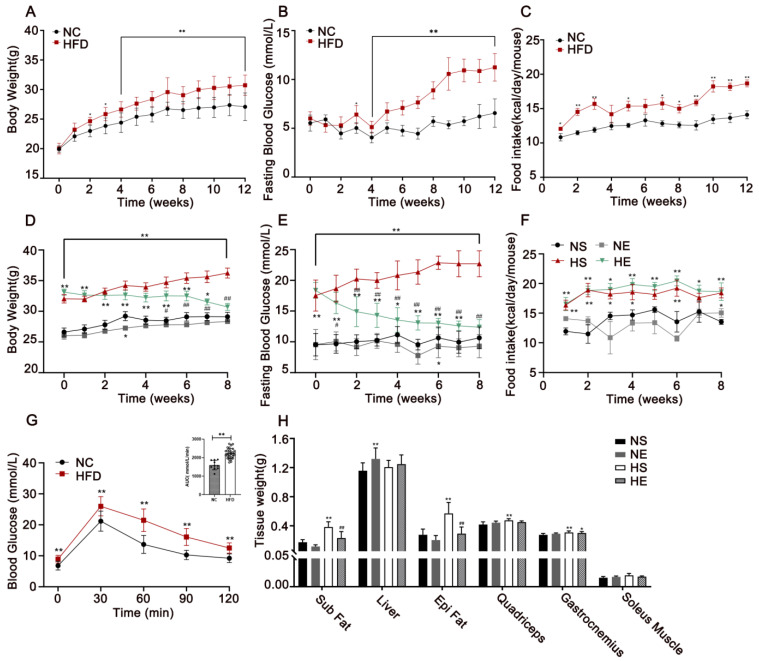
Aerobic exercise attenuated HFD-induced adiposity. (**A**) Body weight, (**B**) fasting blood glucose, and (**C**) food intake during the 12-week feed. (**D**) Weekly changes in body weight, (**E**) fasting blood glucose, and (**F**) food intake during eight weeks of exercise. (**G**) Glucose tolerance test (GTT) and AUC_GTT_ after 12 weeks of the high-fat diet. (**H**) Tissue weights of the mice. NC, normal control, *n* = 20; HFD, high-fat diet, *n* = 30. NS, normal sedentary; NE, normal + exercise; HS, HFD sedentary; HE, HFD + exercise. Sub Fat, subcutaneous fat, Epi Fat, epididymal fat. Values are means ± SD (*n* = 8 per group). * *p* < 0.05, ** *p* < 0.01 vs. NS group; # *p* < 0.05, ## *p* < 0.01 HS group.

During the 12 weeks of HFD, the body weight, fasting blood glucose level, and food intake increased in HFD mice (Figure 2A–C). There was a significant difference in body weight and fasting blood glucose from the fourth week. After 12 weeks of diet intervention, IR was diagnosed if the AUC during GTT of the HFD mice were 1.2 times higher than that of the NC group. The success rate of the IR mice model was 78.57%. After 8 weeks of aerobic exercise, the results showed that exercise had limited the mice’s weight and fasting blood glucose gain compared with the HS group (Figure 2D,E) but had no clear effect on their average food intake (Figure 2F). The decreased body weight of aerobic exercise-treated mice was largely attributable to the reduced mass of white adipose tissue (WAT) deposits, including subcutaneous (*p* = 0.0002), and epididymal fat (*p* < 0.0001). Compared with HS mice, the changes in the liver (*p* = 0.6508) and skeletal muscles (*p* > 0.05) were not significant (Figure 2H). These results suggested that aerobic exercise alleviated HFD-induced adiposity, while that was not associated with muscle mass.

### 3.2. Aerobic Exercise Reduced HFD-Induced IR and Dyslipidemia

**Figure 3:**
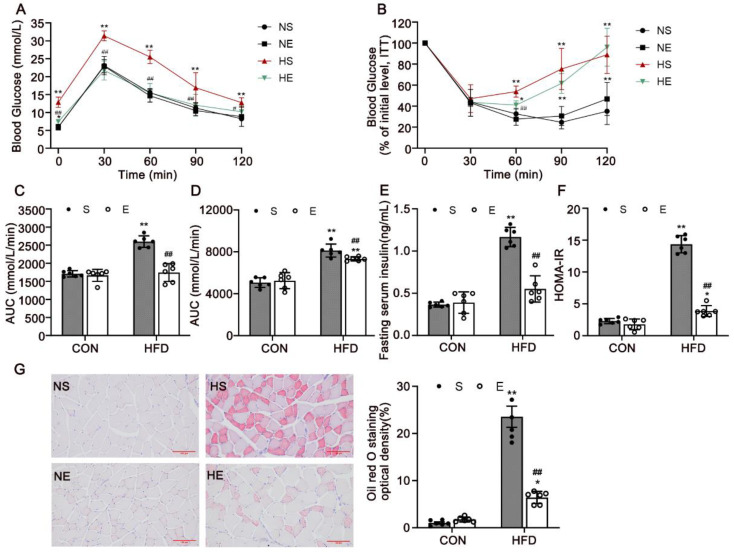
Aerobic exercise improved HFD-induced insulin resistance and dyslipidemia. (**A**) GTT. (**B**) Insulin tolerance test (ITT). (**C**) AUC of GTT. (**D**) AUC of ITT. (**E**) Serum insulin level of mice fasted for 12 h. (**F**) The value of HOMA-IR. (**G**) Oil Red O staining of lipid droplets in skeletal muscle (scale bar = 100 μm). CON, normal control; HFD, high-fat diet; S, sedentary; E, exercise. NS, normal sedentary; NE, normal + exercise; HS, HFD sedentary; HE, HFD + exercise. * *p* < 0.05, ** *p* < 0.01 vs. NS group; # *p* < 0.05, ## *p* < 0.01 HS group.

To investigate whether aerobic exercise could improve IR and glucoregulatory capacity, we measured GTT, ITT, FINS, and HOMA-IR (Figure 3). The blood glucose concentrations in the HS mice were significantly higher than those in the NS group for the duration of the experiment (*p* < 0.0001). Aerobic exercise-treated mice exhibited reduced blood glucose levels at 0 min and 60 min after glucose and insulin challenge (Figure 3A,B; *p* < 0.0001). The GTT and ITT data revealed that glucose intolerance and IR were ameliorated by aerobic exercise. Aerobic exercise significantly decreased the serum insulin levels and HOMA-IR in HFD mice under fasting conditions (Figure 3E,F; *p* < 0.0001).

Moreover, serum TC and TG levels decreased in HE mice in response to exercise (Table 2). Oil Red O staining was used to determine the accumulation of neutral lipids in skeletal muscle (Figure 3G). We found that HE mice were protected from lipid accumulation in muscles compared with HS mice (Figure 3H; *p* < 0.0001). These results indicated that 8 weeks of exercise training may have reduced hyperglycemia, hyperinsulinemia, IR, and dyslipidemia in HFD mice.

### 3.3. Aerobic Exercise Upregulated IRS-1/PI3K/AKT Signaling in Skeletal Muscle

**Figure 4:**
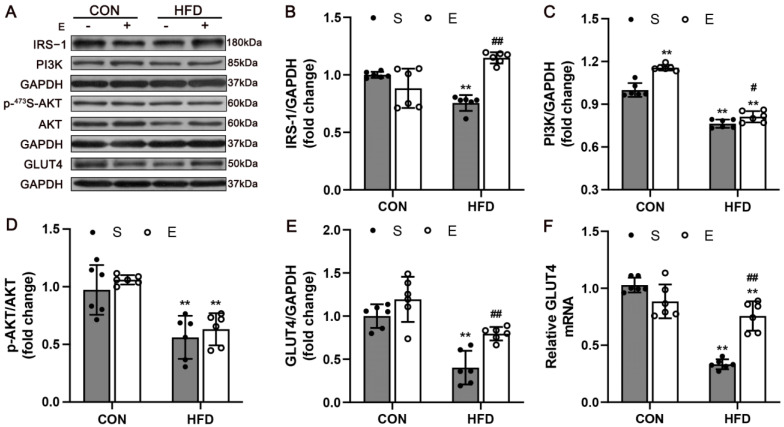
Aerobic exercise attenuated insulin resistance through the upregulation of IRS−-1/PI3K/AKT signaling. (**A**) Western blot images and quantifications of muscle samples, including: (**B**) IRS−-1, (**C**) PI3K, (**D**) p-−AKT/AKT, and (**E**) GLUT4. (**F**) mRNA expression of GLUT4 in skeletal muscle. Values are expressed as the mean ± SD. CON, normal control; HFD, high-fat diet; S, sedentary; E, exercise. ** *p* < 0.01 vs. NS group; # *p* < 0.05, ## *p* < 0.01 HS group (n = 6).

Skeletal muscle glucose uptake occurs due to activation of the IRS-1/PI3K/AKT pathway, and GLUT4 is the major glucose transporter in skeletal muscle [23,24,25]. According to western blot analysis, aerobic exercise improved the protein levels of IRS-1 (*p* < 0.0001), PI3K *(p* = 0.031) and GLUT4 (*p* = 0.0063) (Figure 4B,C,E). Moreover, the levels of AKT and p-AKT expression were determined. The expression of p-AKT in the skeletal muscle was significantly decreased in HS mice (*p* = 0.0013), and the protein levels of p-AKT were slightly increased in the HE mice compared with the HS group (Figure 4D, *p* = 0.8688). In addition, an elevated mRNA expression of GLUT4 was also found (*p* < 0.0001; Figure 4F). The results indicated that exercise remarkably improved glucose uptake in muscle, which might induce activation of the IRS-1/PI3K/AKT signaling pathway to promote the GLUT4 expression and translocation.

### 3.4. Aerobic Exercise Attenuated Skeletal Muscle Inflammation in HFD Mice

**Figure 5:**
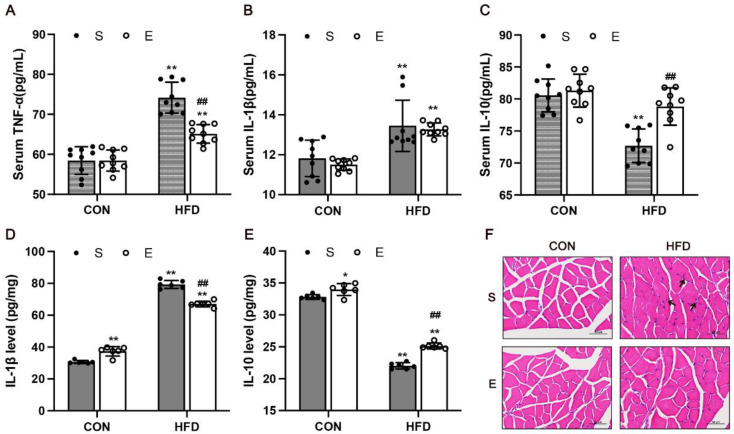
Aerobic exercise attenuated skeletal muscle inflammation. (**A**) TNF-α, (**B**) IL-1β, and (**C**) IL-10 levels in serum by ELISA. (**D**) IL-1β and (**E**) IL-10 levels in skeletal muscle by ELISA. (**F**) Hematoxylin and eosin (H&E) staining of skeletal muscle (scale bar = 50 μm). Black arrows indicate inflammatory infiltration. CON, normal control; HFD, high-fat diet; S, sedentary; E, exercise. NS, normal sedentary; NE, normal + exercise; HS, HFD sedentary; HE, HFD + exercise. * *p* < 0.05, ** *p* < 0.01 vs. NS group; ## *p* < 0.01 HS group. n = 6–9.

Inflammation is associated with skeletal muscle IR. In our study, the results of ELISA illustrated the effects of exercise on TNF-α, IL-1β, and IL-10 levels in the serum of mice with HFD-induced obesity. There were significantly higher levels of TNF-α (*p* < 0.0001) and IL-1β (*p* = 0.001) in the serum of HFD mice compared with the NC group (Figure 5A,B), and aerobic exercise significantly decreased the level of TNF-α (*p* < 0.0001). The level of anti-inflammatory cytokine IL-10 was remarkably increased in the HE mice compared with the HS mice in serum (*p* < 0.0001) (Figure 5C). Meanwhile, the protein levels of IL-1β and IL-10 levels in skeletal muscle of mice were evaluated. We found that the level of IL-1β was significantly decreased (*p* < 0.0001) and the anti-inflammatory cytokine IL-10 was remarkably increased (*p* < 0.0001) in the HE mice compared with the HS mice. (Figure 5D,E). The changes of inflammatory cytokines in skeletal muscle are similar to those in serum.

Furthermore, inflammation of skeletal muscle was evaluated using hematoxylin and eosin (H&E) staining. The results showed many inflammatory cells in the HS group, and exercise alleviated inflammatory infiltration in skeletal muscles of the HFD mice (Figure 5F). Macrophage infiltration is a hallmark of chronic inflammation. Macrophage infiltration is a hallmark of chronic inflammation. These findings revealed that aerobic exercise could decrease inflammatory response in mice with HFD-induced obesity by altering the levels of cytokines and dramatically reducing macrophage infiltration.

### 3.5. Aerobic Exercise Attenuated HFD-Activated NF-κB Signaling and Regulated Inflammatory Cytokines in Skeletal Muscle

**Figure 6:**
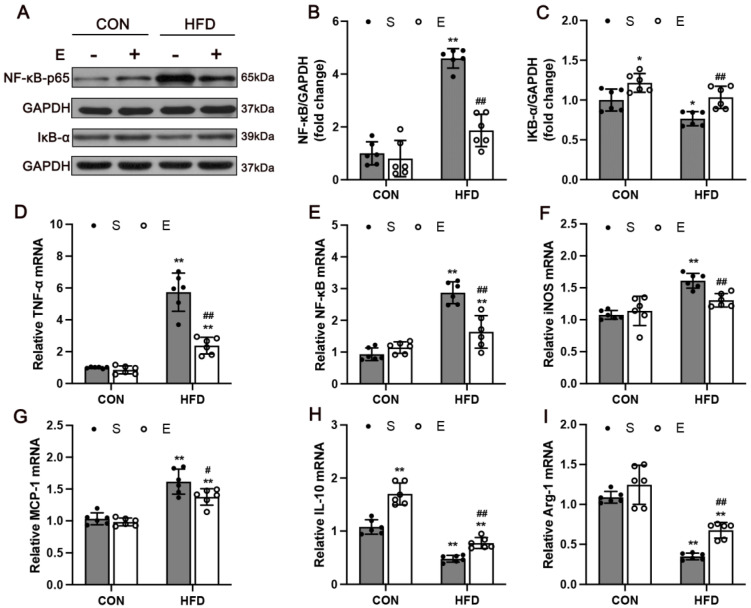
Aerobic exercise ameliorates skeletal muscle inflammation in HFD-fed mice. (**A**) Western blot images and quantifications of muscle samples, including: (**B**) NF-κB and (**C**) IκB-α, n = 6. (**D**) mRNA expression of TNF-α, NF-κB (**E**), iNOS (**F**), MCP-1 (**G**), IL-10 (**H**), Arg-1 (**I**) in skeletal muscle. Values are expressed as the mean ± SD. CON, normal control; HFD, high-fat diet; S, sedentary; E, exercise. * *p* < 0.05, ** *p* < 0.01 vs. NS group; # *p* < 0.05, ## *p* < 0.01 HS group. n = 6.

To understand the mechanism of the NF-κB signaling pathway of the HFD model in exercise training, the mRNA and protein levels of NF-κB of the gastrocnemius of the four groups were studied. As shown in Figure 6B, NF-κB levels were significantly higher in the HS group compared with the NS group (*p* < 0.0001). Aerobic exercise significantly decreased the expression of NF-κB (*p* < 0.0001). Surprisingly, there was a significant increase in the protein expression of IκB-α (Figure 6C; *p* = 0.006). In addition, the data showed that the mRNA expression of NF-κB in the HE group was significantly higher than that in the HS group (Figure 6E; *p* < 0.0001), and aerobic exercise significantly decreased the levels of NF-κB and remarkably increased the protein level of IκB-α (*p* < 0.0001), suggesting that aerobic exercise could reduce skeletal muscle inflammation by the NF-κB signaling pathway.

Growing evidence shows that macrophages also accumulate in skeletal muscle and may constitute the predominant inflammatory cells in skeletal muscle in obesity [26]. To characterize the infiltration of macrophages, we examined the inflammatory and anti-inflammatory factors expression in the skeletal muscle. Proinflammatory markers related to immune cell activation were increased in HFD mice (Figure 6D–F; *p* < 0.0001), while anti-inflammatory markers were reduced in skeletal muscle (Figure 6H,I; *p* < 0.0001). Moreover, aerobic exercise significantly decreased the mRNA levels of TNF-α (*p* < 0.0001), iNOS (*p* = 0.007), and MCP-1(*p* = 0.0212). The level of anti-inflammatory cytokines IL-10 (*p* = 0.0073) and Arg-1 (*p* = 0.0032) were remarkably increased in muscle tissue.

## 4. Discussion

Skeletal muscle insulin resistance is a hallmark of obesity and type 2 diabetes mellitus [27]. Aerobic exercise reduces inflammation and improves insulin resistance in many tissues, including skeletal muscle [28]. In this study, we demonstrated that 8 weeks of aerobic exercise mitigated HFD-induced weight gain, insulin resistance, and chronic inflammation in the skeletal muscle of mice. Moreover, aerobic exercise attenuated insulin resistance by regulating the IRS-1/PI3K/AKT and the TNF-α/NF-κB signaling pathways.

Consistent with previous reports [29,30], we showed that exercise could improve systemic glucose tolerance and insulin sensitivity in HFD mice. The IRS-1/PI3K/AKT pathway is one of the main regulatory pathways of IR and has integrative roles in glucose and lipid metabolism [31]. Skeletal muscle glucose uptake occurs when insulin acts via the IR and IRS-1 to phosphorylate and thereby activate AKT, and consequent translocation of the insulin-sensitive glucose transporter GLUT4 to the sarcolemma occurs [25,32]. Aerobic exercise is an effective way to stimulate GLUT4 expression in skeletal muscle. According to our study, although there were no significant differences found in the protein expression in the phosphorylation of AKT protein between HE mice and control mice, inhibited IRS-1/PI3K/AKT pathway and GLUT4 protein content were observed in the skeletal muscles of the HFD mice, and aerobic exercise reversed the inactivation of the pathway. Taken together, these results suggest that aerobic exercise ameliorates insulin resistance in skeletal muscle and that the IRS-1/PI3K/AKT/GLUT4 pathway plays a role in glucose metabolism.

The improved insulin sensitivity in the HE mice was accompanied by decreased inflammation in skeletal muscles. Obesity and IR are highly associated with inflammatory responses and cytokines [33,34]. Obesity or ectopic fat accumulation induces an innate immune response with subsequent recruitment of immune cells, which ultimately leads to the development of IR [35,36,37]. Macrophage infiltration and phenotypes changing are the main characteristics of obesity-linked inflammation [14,38]. TNF-α has been shown to promote insulin resistance in different ways [39,40]. There is evidence indicating that treatment with anti-TNF antibodies improves hepatic IR [41]. MCP-1 increase early in skeletal muscle and visceral AT of mice fed a HFD, and the increase in MCP-1 appears to precede the increase in macrophages and the activation of cytokines [42]. TNF-α and iNOS are crucial for macrophage polarization into M1 proinflammatory phenotypes, while IL-10 and Arg-1 can alter activated (M2) phenotypes. Under stimulation, adipocytes and myocytes secrete more chemokines, which induce immune cell migration. For better visibility of the situation of macrophage polarization in skeletal muscle, macrophages should be identified by direct immunofluorescence staining in future studies. Moreover, NF-κB is an inflammatory pathway associated with IR and obesity [43]. Consistent with relevant studies, we found that the HFD mice exhibited increased TNF-α and NF-κB levels in serum and muscle tissues.

Exercise can modulate the inflammatory response by regulating specific inflammatory signaling pathways that can interfere with the signaling pathways of glucose uptake [44,45]. Our findings are consistent with the anti-inflammatory effects of exercise and its preventive effect on IR [46,47,48]. Here, we found that aerobic exercise regulated inflammatory factors, including significantly decreased proinflammatory cytokines and increased levels of anti-inflammatory factors in the skeletal muscle, suggesting that exercise may reduce the infiltration of macrophages thereby reducing inflammation. In the classical pathway, TNF-α activates IκB kinase (IKK) and phosphorylates IKKβ, and induces degradation of IκB-α, thereby resulting in the nuclear translocation of NF-κB and inducing an inflammation-related transcription process [49]. Exercise can inhibit NF-κB, leading to decreased expression of inflammatory proteins [50,51]. In our study, aerobic exercise significantly reduced NF-κB mRNA and protein expression in skeletal muscles. Few studies have explored the changes in IκB-α expression due to exercise. To better interpret the exercise-induced inflammatory response, further studies should evaluate IκB-α phosphorylation changes. NF-κB is also a downstream molecule of the PI3K/AKT signaling pathway [52]. The activation of IKK can also phosphorylate IRS-1 at serine residue, thereby impeding tyrosine phosphorylation and causing IR [53]. Therefore, the improvement of IR by exercise is probably due to the reduction of inflammation. In skeletal muscle, the NF-κB pathway may also be important for adaptation to aerobic exercise training.

## 5. Conclusions

In conclusion, our findings demonstrated that aerobic exercise attenuated HFD-related IR in skeletal muscle, which may be associated with the reduced TNF-α/NF-κB signaling and activated IRS-1/PI3K/AKT pathway. We also found aerobic exercise decreased the number of and altered phenotypes of macrophages in skeletal muscle, which needs more evidence in further studies. The present study may provide a basis for a new therapeutic strategy for IR. Further studies are needed to fully understand the specific mechanism of the NF-κB pathway in chronic inflammation in skeletal muscle.

## Figures and Tables

**Table 1:** Primer sequences.

Gene	Primer Sequences (5′-3′)		Product Size
TNF-α	Forward Primer	CCCTCACACTCAGATCATCTTCT	199 bp
	Reverse Primer	GCTACGACGTGGGCTACAG	
MCP-1	Forward Primer	AAGAAGGAATGGGTCCAGACA	140 bp
	Reverse Primer	GCTTCAGATTTACGGGTCAACT	
iNOS	Forward Primer	TCCATGCTAATGCGAAAGG	197 bp
	Reverse Primer	CTTGTCACCACCAGCAGTAGTT	
Arg-1	Forward Primer	ACAGCAGAGGAGGTGAAGAGTAC	100 bp
	Reverse Primer	AGTCAGTCCCTGGCTTATGGT	
IL-10	Forward Primer	TTGCCAAGCCTTATCGGA	103 bp
	Reverse Primer	ACCCAGGGAATTCAAATGC	
NF-κB	Forward Primer	AGGACCTATGAGACCTTCAAGAGTA	145 bp
	Reverse Primer	GGAAGGTGTAGGGCTGCG	
GLUT4	Forward Primer	TTGGCTCCCTTCAGTTTGG	233 bp
	Reverse Primer	CCTTTTCCTTCCCAACCATT	
β-actin	Forward Primer	GTGCTATGTTGCTCTAGACTTCG	174 bp
	Reverse Primer	ATGCCACAGGATTCCATACC	

**Table 2:** Effects on metabolic parameters after eight weeks of aerobic exercise.

Blood Lipids (mmol/L)	NS	NE	HS	HE	*p* Value
TC	2.62 ± 0.43	3.02 ± 0.53	4.67 ± 0.40 **	3.89 ± 0.70 *^,##^	HS vs. NS *p* < 0.0001 HE vs. HS *p* = 0.007 HE vs. NS *p* = 0.041
TG	0.59 ± 0.07	0.60 ± 0.09	0.88 ± 0.19 **	0.72 ± 0.07 *^,##^	HS vs. NS *p* < 0.0001 HE vs. HS *p* = 0.008 HE vs. NS *p* = 0.017
HDL	3.14 ± 0.59	3.21 ± 0.55	3.75 ± 0.73 *	3.55 ± 0.24	HS vs. NS *p* =0.033 HE vs. HS *p* = 0.465 HE vs. NS *p* = 0.275
LDL	0.15 ± 0.08	0.14 ± 0.06	0.32 ± 0.13 **	0.31 ± 0.13	HS vs. NS *p* = 0.003 HE vs. HS *p* = 0.891 HE vs. NS *p* = 0.894

Data are presented as mean ± SD, n = 8. * *p* < 0.05 vs. NS; ** *p* < 0.01 vs. NS; ^##^ *p* < 0.01 vs. HS. NS, normal control + sedentary; NE, normal control + exercise; HS, high-fat diet + sedentary; HE, high-fat diet + exercise; TG, triglycerides; TC, total cholesterol; HDL, high-density lipoprotein; LDL, low-density lipoprotein.

## Data Availability

The authors confirm that the data supporting the findings of this study are available within the article.
